# Supplementary material for: Prediction of coronary heart disease incidence in a general male population by circulating non-coding small RNA sRNY1-5p in a nested case–control study
Source: Sci Rep. 2021 Jan 19;11:1837. doi: 10.1038/s41598-021-81221-8 (PMC7815790; doi:10.1038/s41598-021-81221-8)
Supplement: Supplementary file 4 — Supplementary Information 4. [file 41598_2021_81221_MOESM4_ESM.pdf]

Supplemental Table 4. Primer sequences

	Forward primer	Reverse primer
human s-RNY1-5p (stem-loop RT primer)		5’- GTCGTATCCAGTGCAGGGTCCGAGGTATTCGCACTGGATACGACATTGAG -3’
universal primer (qPCR)		5’- GTGCAGGGTCCGAGGT -3’
human s-RNY1-5p (qPCR)	5’- TGGTCCGAAGGTAGTGAGT -3’	
