# Supplementary figures and images for: Prediction of coronary heart disease incidence in a general male population by circulating non-coding small RNA sRNY1-5p in a nested case–control study
Source: Sci Rep. 2021 Jan 19;11:1837. doi: 10.1038/s41598-021-81221-8 (PMC7815790; doi:10.1038/s41598-021-81221-8)

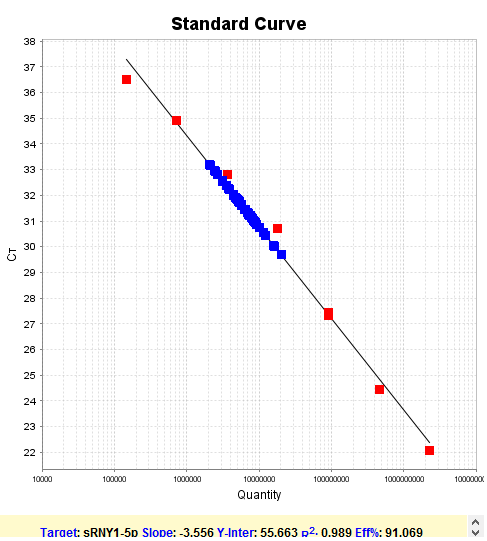

Supplement: Supplementary file 5 — Supplementary Information 5. [file 41598_2021_81221_MOESM5_ESM.tif]
